# The Role of Perivascular Adipose Tissue in Non-atherosclerotic Vascular Disease

**DOI:** 10.3389/fphys.2017.00969

**Published:** 2017-11-28

**Authors:** Tetsuo Horimatsu, Ha Won Kim, Neal L. Weintraub

**Affiliations:** Division of Cardiology, Department of Medicine, Vascular Biology Center, Medical College of Georgia at Augusta University, Augusta, GA, United States

**Keywords:** perivascular adipose tissue, neointimal formation, aortic aneurysm, arterial stiffness, vasculitis

## Abstract

Perivascular adipose tissue (PVAT) surrounds most large blood vessels and plays an important role in vascular homeostasis. PVAT releases various chemokines and adipocytokines, functioning in an endocrine and paracrine manner to regulate vascular signaling and inflammation. Mounting evidence suggests that PVAT plays an important role in atherosclerosis and hypertension; however, the role of PVAT in non-atherosclerotic vascular diseases, including neointimal formation, aortic aneurysm, arterial stiffness and vasculitis, has received far less attention. Increasing evidence suggests that PVAT responds to mechanical endovascular injury and regulates the subsequent formation of neointima via factors that promote smooth muscle cell growth, adventitial inflammation and neovascularization. Circumstantial evidence also links PVAT to the pathogenesis of aortic aneurysms and vasculitic syndromes, such as Takayasu's arteritis, where infiltration and migration of inflammatory cells from PVAT into the vascular wall may play a contributory role. Moreover, in obesity, PVAT has been implicated to promote stiffness of elastic arteries via the production of reactive oxygen species. This review will discuss the growing body of data and mechanisms linking PVAT to the pathogenesis of non-atherosclerotic vascular diseases in experimental animal models and in humans.

## Key concepts

- Accumulating data suggest that PVAT plays an important role not only in atherosclerosis and hypertension, but also in non-atherosclerotic vascular diseases.
- Phenotypic changes in PVAT after vascular injury promote release of adipocytokines that can regulate inflammation, VSMC proliferation and neovascularization, thereby contributing to neointimal formation.
- Phenotypic changes in PVAT in response to high fat diet or smoking may promote vascular inflammation, ROS production, VSMC phenotype switching and matrix degradation to augment AAA formation.
- Inflamed PVAT is associated with arterial stiffness and vasculitis; however, direct evidence of a pathologic role of PVAT is lacking.
- Much of the available data linking PVAT to non-atherosclerotic vascular diseases is associative rather than direct in nature due to the challenges in developing specific experimental models to test the impact of PVAT on these disease states.

## Introduction

Perivascular (PV) adipose tissue (PVAT) surrounds most large blood vessels except the cerebral vasculature, juxtaposed to the vascular adventitia and devoid of an anatomical barrier. The absence of an anatomic barrier suggests that mediators such as adipokines and cytokines released from PVAT can readily gain access into the blood vessel wall. Traditionally, PVAT had been thought to simply provide structural support for blood vessels; however, over the past two decades, it has become recognized as a physiologically and metabolically active endocrine tissue with important effects on vascular function and disease (Verhagen and Visseren, 2011; Fitzgibbons and Czech, 2014).

The bulk of adipose tissue present in adult animals is white adipose tissue (WAT) contained in visceral and subcutaneous depots, which is designed for energy storage and mobilization. By contrast, depending on the anatomic location, PVAT exhibits features of both white and brown adipose tissue (BAT), the latter of which is specialized for thermogenic energy expenditure (Brown et al., 2014). Mature adipocytes within visceral and subcutaneous adipose depots are thought to originate from precursor cells with distinct embryologic lineages (Tchkonia et al., 2007), and PV adipocytes may be distinct from other adipocytes by virture of their putative origin from vascular smooth muscle cell (VSMC) progenitors (Cai et al., 2011; Chang et al., 2012). Although adult human coronary PVAT exhibits the morphology of WAT, the PV adipocytes contained in this depot are more heterogeneous in shape and smaller in size and exhibit a reduced state of adipogenic differentiation as compared with adipocytes residing in subcutaneous and perirenal adipose depots derived from the same subjects (Chatterjee et al., 2009). Like other adipose tissues, human coronary PVAT secretes both pro-inflammatory and anti-inflammatory adipocytokines and chemokines; however, the balance is strongly shifted toward inflammation via elevated secretion of cytokines such as interleukin (IL)-6, IL-8, and in particular, monocyte chemoattractant protein (MCP)-1, concomitant with reduced secretion of anti-inflammatory adiponectin (Chatterjee et al., 2009). In rodents, the gene expression profile of thoracic aortic PVAT is similar to that of BAT (Fitzgibbons et al., 2011; Padilla et al., 2013), while PVAT surrounding the abdominal aorta contains a mixture of WAT and BAT; in contrast, mesenteric, carotid, and femoral PVAT exhibit a purely WAT phenotype. These findings suggest that PVAT exhibits considerable phenotypic heterogeneity depending on its anatomic location.

Given these unique features of PV adipocytes and PVAT, there is great interest in understanding their role in vascular function and disease. In animal models, PVAT has been reported to possess both protective and detrimental effects on vascular function, depending on the experimental model and associated pathological states such as obesity and metabolic disease, which impact the production and/or bioactivity of vasoactive factors and inflammatory mediators. Mounting evidence also suggests that dysfunctional PVAT plays an important role in atherosclerosis and hypertension, in part by promoting insulin resistance and metabolic disease (Eringa et al., 2007). On the other hand, the brown-like function of PVAT can elicit favorable metabolic effects associated with non-shivering thermogenesis and combustion of fatty acids to ameliorate atherosclerosis (Chang et al., 2012). Recent emerging data suggest that PVAT may also modulate non-atherosclerotic vascular diseases, including neointimal formation, aortic aneurysm, arterial stiffness and vasculitis. It is important to point out that much of the available data in non-atherosclerotic diseases is associative rather than direct in nature due to the challenges in developing specific experimental models to test the impact of PVAT on these disease states. This article will review the available evidence and putative mechanisms linking PVAT and non-atherosclerotic vascular diseases.

## PVAT and neointimal formation

### Pathogenesis of neointimal formation

Neointimal formation is initiated by mechanical injury to the arterial endothelium, followed by local inflammatory cell recruitment, production of chemokines and growth factors that promote migration of VSMCs and adventitial fibroblasts into the intimal layer, and neovascularization (Goel et al., 2012). VSMCs robustly proliferate in the intima and deposit extracellular matrix, in a process analogous to scar formation. PVAT releases a variety of adipokines and cytokines that potentially can regulate multiple steps of neointimal formation. In lean mice, adiponectin produced by PVAT exerts anti-inflammatory effects to attenuate neointimal formation (Matsuda et al., 2002). In contrast, Wang et al. reported that visfatin, a PVAT-produced adipocytokine with important effects on glucose metabolism, stimulates VSMC proliferation (Wang et al., 2009). Leptin is an adipocyte-derived hormone with pro-inflammatory effects whose expression in PVAT is increased in obesity. Using an adenoviral vector, Schroeter et al. locally overexpressed leptin in the carotid artery after vascular injury and detected a significant increase in luminal stenosis and the intima-to-media ratio (serum leptin levels were similar among the groups) (Schroeter et al., 2013). The investigators further demonstrated that recombinant human leptin significantly increased VSMC proliferation *in vitro*, suggesting that PVAT could promote neointimal formation through local leptin production. PVAT-derived leptin was also associated with VSMC phenotypic switching to a synthetic phenotype via activation of the p38 mitogen-activated protein kinase (MAPK) signaling pathway (Shin et al., 2005; Li et al., 2014). Like leptin, expression of the pro-inflammatory chemokine MCP-1 is upregulated in PVAT in obesity, and MCP-1 also promotes VSMC proliferation (Aiello et al., 1999). The role of MCP-1 released from PVAT in neointimal formation will be discussed in depth in section Experimental Evidence Demonstrating That PVAT Can Modulate Neointimal Formation.

Endothelial dysfunction is associated with reduced nitric oxide (NO) production through impaired endothelial NO synthase (eNOS) activity, and eNOS gene deletion promoted inward vascular remodeling and enhanced neointimal formation after external carotid artery ligation (Rudic et al., 1998). Furthermore, eNOS deletion induced the expression of stromal cell-derived factor-1α, which plays an important role in recruitment of VSMC progenitor cells into the neointima (Zhang et al., 2006). PVAT was reported to induce endothelial dysfunction via protein kinase C-β dependent phosphorylation and inactivation of eNOS (Payne et al., 2009). PVAT also may inhibit endothelial NO production through increased expression of caveolin-1, which negatively regulates eNOS via interruption of calcium/calmodulin signaling (Lee et al., 2014). These data suggest that PVAT can promote endothelial dysfunction by impaired NO signaling, thus contributing to neointimal formation.

Adventitial fibroblasts and myofibroblasts directly migrate into the arterial neointima after endovascular injury. Ruan et al. investigated the role of PVAT-derived factors in the regulation of adventitial fibroblast activation and demonstrated that PVAT-derived complement 3 is required for adventitial fibroblast migration and adventitial remodeling in deoxycorticosterone acetate-salt hypertensive rats (Ruan et al., 2010). This suggests an important role of PVAT in promoting fibroblast activation and migration during neointimal formation.

Adventitial neovascularization is associated with vascular restenosis after balloon arterial injury. These neovessels can serve as conduits for inflammatory cell trafficking into the injured blood vessel, and rupture of the neovessels can potentially promote intraplaque hemorrhage leading to acute vascular occlusion (Chen et al., 2016). Human coronary PV adipocytes were demonstrated to secrete higher levels of biologically active pro-angiogenic factors than subcutaneous adipocytes, suggesting a potential role in regulating neovascularization following vascular injury (Chatterjee et al., 2013). Indeed, PV transplantation of epididymal adipose tissue to the ligated carotid artery in mice resulted in a 3 fold increase in *vasa vasorum* density, implying a potential role for PVAT in promoting plaque neovascularization (Tanaka et al., 2015). In humans, the release of pro-angiogenic vascular endothelial growth factor (VEGF) from PVAT of type 2 diabetics was significantly higher than that from PVAT of non-diabetic subjects or from subcutaneous adipose tissue of diabetics (Schlich et al., 2013). Moreover, conditioned medium from PVAT of type 2 diabetic patients induced potent effects on VSMC proliferation, suggesting that dysfunctional PVAT could play an important role in promoting both neovascularization and vascular restenosis in diabetic patients.

### Effects of mechanical injury on PVAT

While endovascular injury originates in the endothelium, its impact is felt throughout the blood vessel wall, including the adventitia and PV adipocytes. Indeed, mechanical arterial injury induces histological and phenotypic changes locally in PVAT surrounding the injured artery (Okamoto et al., 2001; Rajsheker et al., 2010; Takaoka et al., 2010). Inflammatory leukocytes were detected in PVAT just 1 day after balloon injury in pig coronary arteries, and mRNA expression of vascular cell adhesion molecule (VCAM)-1 was increased in PVAT at 3 days after injury (Okamoto et al., 2001). In the rat wire injury model, F4/80-positive macrophages and CD3-positive T cells were observed to accumulate in PVAT after endovascular injury. In addition, MCP-1 and IL-6 were upregulated in PVAT 1 day after endovascular injury, which was attenuated in tumor necrosis factor (TNF)-α knockout mice (Takaoka et al., 2010), suggesting that mechanical vascular injury is associated with activation of discreet inflammatory pathways in PVAT.

Interestingly, inflammatory changes in PVAT in humans may be detectable using computerized tomography (CT) imaging (Antonopoulos et al., 2017). In this study, the authors demonstrated that the degree of coronary PVAT inflammation correlated with the fat attenuation index (FAI), reflecting changes in the balance between lipid and aqueous phase due to alterations in adipocyte size and lipid content. FAI in PVAT distinguished vulnerable atheromatous plaques causing acute myocardial infarction from stable or previously stented plaques. These findings suggest that biological and phenotypic characteristics of PVAT can change dynamically in response to spontaneous lesion destabilization and mechanical injury. Moreover, PV FAI may be a promising noninvasive method to dynamically monitor vascular inflammation and the extent of underlying vascular disease.

### Caveats associated with experimental models employed to test the role of PVAT in neointimal formation

To test the role of PVAT in vascular disease, animal models that spontaneously lack PVAT merit consideration, including the A-ZIP/F-1 mouse, the FATATTAC mouse, and the SMPG knockout mouse (Moitra et al., 1998; Pajvani et al., 2005; Chang et al., 2012). Amongst these models, only the SMPG knockout mouse (generated by breeding PPARγ-floxed mice with SM22α-Cre mice) is selectively devoid of PVAT rather than generally lipodystrophic. However, loss of PPARγ expression in VSMCs in these mice may complicate the vascular phenotype (Chang et al., 2012). Surgical models involving removal of endogenous PVAT and/or transplantation of adipose tissue to the arterial wall have also been developed (Takaoka et al., 2009; Öhman et al., 2011; Tian et al., 2013). However, these surgical models pose major technical challenges given the minute size of this adipose depot in mice, the potential for non-specific arterial injury, and/or confounding systemic metabolic effects if a large enough quantity of fat is transplanted. Moreover, most studies have transplanted subcutaneous or epididymal fat, which differs phenotypically from PVAT, to the vascular wall (Takaoka et al., 2009; Öhman et al., 2011; Tian et al., 2013). These technical and methodological nuances must be carefully considered when drawing conclusions from experimental studies probing the role of PVAT in neointimal hyperplasia and other vascular diseases.

### Experimental evidence demonstrating that PVAT can modulate neointimal formation

The impact of adipose tissue removal and transplantation on neointimal formation has been examined in experimental animal models using either PVAT (directly) or subcutaneous/visceral adipose tissue (indirectly). Takaoka et al. investigated the impact of diet-induced obesity on inflammatory responses in PVAT and its subsequent role in the development of neointimal formation (Takaoka et al., 2009). The investigators performed femoral wire injury in mice in the presence or absence of endogenous PVAT. Then, 10 mg of visceral (epididymal) or subcutaneous fat tissue was harvested from wild-type mice fed a chow diet or high fat/high sucrose diet and transplanted around the wire-injured femoral artery. Notably, removal of PVAT enhanced neointimal formation after vascular wire injury, which was attenuated by transplantation of subcutaneous adipose tissue from mice fed a normal chow diet. Transplanting subcutaneous adipose tissue from adiponectin-deficient mice significantly enhanced lesion formation, which was abrogated by local application of recombinant adiponectin to the periadventitial area (Matsuda et al., 2002). In contrast, transplanting epididymal fat, or subcutaneous fat from mice fed a high fat/high sucrose diet, failed to attenuate neointimal formation. These data suggest that periadventitial fat can protect against neointimal formation after angioplasty under physiological conditions through production of adiponectin; the extent to which surgical manipulation vs. removal of PVAT *per se* augmented wire injury, however, is unclear.

Tian et al. reported that PVAT-derived angiopoietin-like protein (Angptl) 2, an adipose tissue-derived pro-inflammatory factor, augments neointimal formation after endovascular injury (Tian et al., 2013). In this study, 10 mg of epididymal adipose tissue was harvested from Angptl2 knockout mice or wild-type mice and transplanted around the wire-injured femoral arteries (note that the expression of Angptl2 in visceral adipose tissue was reportedly comparable with that in PVAT surrounding the femoral artery). Compared with transplantation of adipose tissue from wild-type mice, transplantation of adipose tissue from Angptl2-deficient mice led to decreased neointimal formation following endovascular injury. Expression of TNF-α and IL-1β in adipose tissue from Angptl2 knockout mice was significantly decreased, in conjunction with fewer accumulating macrophages. In addition, vascular matrix metalloproteinase (MMP)-2 activity was diminished by transplanting adipose tissue from Angptl2 knockout mice. Conversely, transplantation of visceral adipose tissue harvested from transgenic mice overexpressing Angptl2 aggravated neointimal formation in response to endovascular injury, in conjunction with increased inflammatory marker expression and MMP-2 activity. While these findings suggest that Angptl2 expression in transplanted PVAT promotes neointimal formation by augmenting adipose tissue inflammation and extracellular matrix degradation, the transplanted adipose tissues were subcutaneous or visceral in origin, and thus may not directly inform the role of PVAT.

Manka et al. provided direct evidence that PVAT contributes to the vascular response to wire injury, at least in part through an MCP-1-dependent mechanism (Manka et al., 2014). Transplanting PVAT (2-3 mg) from wild-type mice to the carotid artery (which is normally devoid of PVAT) exacerbated neointimal formation following wire injury. The transplanted PVAT augmented accumulation of VSMCs in the neointima and promoted adventitial inflammation and angiogenesis. Notably, transplantation of subcutaneous adipose tissue had no effect in this model. Interestingly, the enhanced neointimal formation and adventitial angiogenesis, but not the adventitial inflammation, were prevented by transplanting PVAT from MCP-1 knockout mice. These findings imply an important role for MCP-1 produced by PVAT in neointimal formation and suggest that molecular imaging of MCP-1 in PVAT could be a promising strategy to quantify the risk of restenosis following arterial injury. Moreover, therapeutically targeting MCP-1 in PVAT might be an effective strategy to prevent restenosis in selected patients.

### Summary of the role of PVAT in neointimal formation

**Figure 1:**
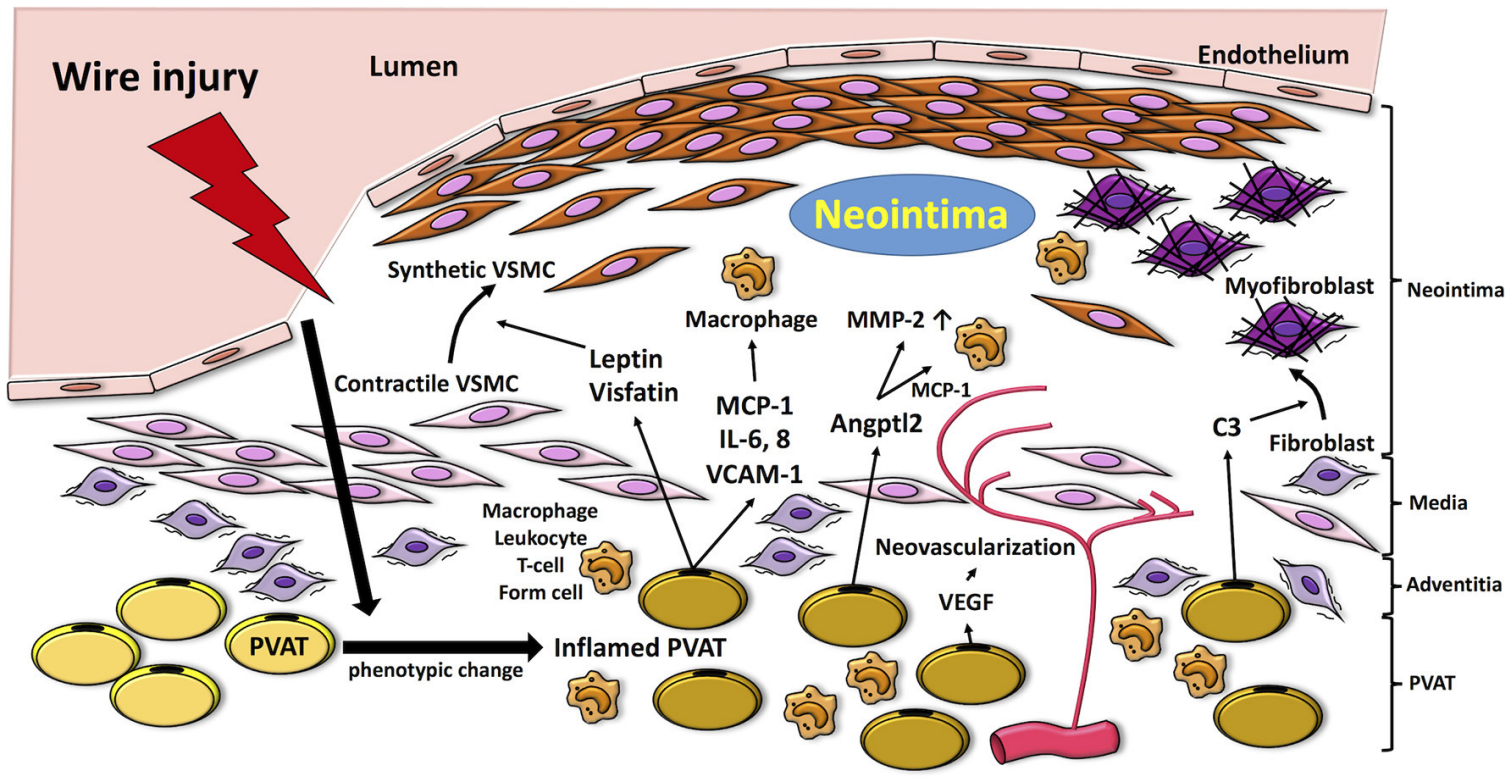
Proposed mechanisms by which PVAT modulates pathways linked to the pathogenesis of neointimal formation. Vascular injury induces inflammation in PVAT, which produces adipocytokines such as MCP-1, IL-6, and VCAM-1 and fosters recruitment of inflammatory cells (i.e., macrophages, neutrophils, T cells) into the vascular adventitia. Release of adipocytokines and chemokines, such as leptin and visfatin, from inflamed PVAT enhances phenotype switching of VSMCs, which proliferate and migrate to the neointima. Moreover, PVAT-medicated Angptl2 elevates MMP-2, and MCP-1 expression. PVAT-derived complement 3 induces adventitial fibroblast migration to neointima. VEGF released from PVAT also contributes to adventitial neovascularization. MCP-1, monocyte chemoattractant protein-1; IL-6, interleukin-6; VCAM-1; vascular cell adhesion molecule-1; VEGF, vascular endothelial growth factor; MMP-2, matrix metalloprotenease-2; VSMC, vascular smooth muscle cell; Angptl2, angiopoietin-like protein 2; C3, complement 3.

PVAT can modulate numerous pathways linked to the pathogenesis of neointimal formation (Figure 1). Accumulating data suggest that phenotypic changes in PVAT occur soon after vascular injury, thereby leading to changes in expression and release of various inflammatory chemokines, cytokines and adipokines that can regulate inflammation, VSMC proliferation, neovascularization, etc. Experimental approaches employing PVAT removal and/or adipose tissue transplantation *in vivo* have been developed to directly or indirectly examine the role of PVAT in neointimal formation. The available data suggest that PVAT can either modulate or mediate neointimal formation, depending on the particular model and underlying metabolic state. However, the current experimental models are technically challenging and have limitations, so our understanding of the role of PVAT in regulating neointimal formation remains incomplete.

## PVAT and aortic aneurysm

### Pathogenesis of abdominal aortic aneurysms (AAAs)

AAAs account for over 13,000 deaths annually in the United States and about 25,000 AAA repairs are performed each year (Thompson, 2002). Only ~25% of patients with aortic rupture survive to surgery, with an additional 50% mortality following surgery (Lederle et al., 2002). AAAs are characterized by localized structural deterioration of the aortic wall, leading to progressive aortic dilation and rupture. Key pathological features of AAAs include pronounced inflammatory cell infiltration, oxidative stress, MMP activation, and VSMC apoptosis and phenotype switching, which cumulatively lead to degradation of extracellular matrix, loss of structural integrity, and weakening of the aortic wall (Kuivaniemi et al., 2015). The inflammation in AAAs extends throughout the vessel wall to the adventitia, which has raised interest in the potential role of PVAT in AAA pathogenesis. Smoking, the most significant risk factor for AAAs (Vardulaki et al., 2000), has been shown to accentuate the pro-inflammatory status of PVAT by enhancing expression and activity of the P2X_7_R-inflammasome complex (Rossi et al., 2014). Wang et al. investigated the levels of inflammatory adipokines produced by PVAT-derived mature adipocytes cultured alone or under nicotine stimulation. Nicotine reduced adiponectin secretion while activating pro-inflammatory nuclear factor (NF)-kB and pro-inflammatory cytokine expression (Wang et al., 2016), further suggesting a link between smoking, PVAT inflammation and AAA pathogenesis.

MMP production by inflammatory cells and VSMCs is fundamental to the pathogenesis of AAAs (Busuttil et al., 1982; Brophy et al., 1991; Kuivaniemi et al., 2015). As mentioned previously, Angptl2 deficiency in transplanted PVAT was reported to attenuate vascular MMP-2 activity and extracellular matrix degradation (Tian et al., 2013). Kurobe et al. also demonstrated that MMP-2 expression in PVAT was significantly decreased by systemic treatment with eplerenone, a selective mineralocorticoid receptor antagonist, which concomitantly inhibited AAA formation (Kurobe et al., 2013). These studies suggest that PVAT-derived MMP-2 could potentially play a role in AAA pathogenesis or progression.

In healthy blood vessels, VSMCs are quiescent and exhibit a contractile or differentiated phenotype, characterized by high level expression of differentiation markers such as α-smooth muscle actin (SMA) and smooth muscle myosin heavy chain (SM-MHC). During pathogenic conditions, VSMCs exhibit phenotype switching, characterized by increased proliferation, decreased expression of differentiation markers, and dysregulated synthesis of extracellular matrix components. Phenotypic switching of VSMCs in the context of elastase-induced aortic aneurysms in rats was reported (Ailawadi et al., 2009; Mao et al., 2015). As mentioned previously, Li et al. showed that PVAT-derived leptin promotes VSMC phenotypic switching through the p38 MAPK-dependent pathway in rats with metabolic syndrome (Li et al., 2014), suggesting that the paracrine action of PVAT-derived adipokines could potentially regulate AAA pathogenesis by promoting VSMC phenotypic switching.

### Anatomic and biological changes in PVAT associated with AAA

Obesity is a risk factor for AAA (Cronin et al., 2013). Like other adipose depots, PVAT expands and becomes more pro-inflammatory during obesity. The quantity of PVAT around the thoracic and abdominal aorta, assessed by CT imaging, was reported to be positively associated with aneurysm diameter in the Framingham Heart Study (Thanassoulis et al., 2012), supporting the notion that local fat depots may contribute to aortic remodeling in human AAAs. Folkesson et al. studied the characteristics of PVAT adjacent to human AAAs in patients undergoing elective surgical repair (Folkesson et al., 2017). They demonstrated that AAAs are surrounded by abundant PVAT enriched in inflammatory cells (neutrophils, macrophages, mast cells, and T cells) and proteases (cathepsin K and S). Moreover, pro-inflammatory IL-6 expression was increased in PVAT by 4-fold as compared to intima/media of the AAA tissues. These data suggest that PVAT could contribute to inflammation in the adjacent aneurysmal aortic wall.

PVAT, like other adipose depots, is richly endowed with mesenchymal stem cells contained in the stromovascular fraction. Stem cells isolated from adipose tissues and implanted into experimentally-generated AAAs in pigs were demonstrated to stabilize the aneurysm, promote fibrosis, blunt inflammation, and induce elastin fiber regeneration (Riera del Moral et al., 2015). Whether the endogenous mesenchymal stem cells contained in PVAT could produce beneficial effects in AAAs is unknown and worthy of investigation. Nevertheless, this observation raises the possibility that PVAT could play a complex and multi-faceted role in modulating AAAs.

### Experimental evidence that PVAT can modulate AAAs

To elucidate the mechanisms of AAA formation, several animal models [i.e., chronic infusion of angiotensin II (AngII), local elastase infusion, and adventitial exposure of calcium chloride] are widely employed (Daugherty and Cassis, 1999; Chiou et al., 2001; Trachet et al., 2015) which exhibit features similar to human AAAs, including inflammatory cell infiltration, VSMC apoptosis and elastin fragmentation. Using the AngII model of AAA formation, Police et al. reported that PVAT surrounding the abdominal aorta exhibited increased numbers of F4/80 positive macrophages and expression of MCP-1 and its receptor, CCR2, as compared to that surrounding the thoracic aorta (Police et al., 2009). Co-localization of PVAT inflammation with AAAs in this model suggests a contributory role in AAA pathogenesis, although this was not directly tested in the study. Gao et al. (2012) reported that high fat diet feeding resulted in endothelial nitric oxide synthase (eNOS) uncoupling in PVAT in obese mice in conjunction with AAA formation, further suggesting a role for obesity-related PVAT dysfunction in the pathogenesis of AAAs.

The recruitment of monocytes to the adventitia, and their subsequent differentiation into CD14-expressing macrophages by IL-6, plays a critical role in aortic aneurysm pathogenesis (Tieu et al., 2009). Notably, soluble CD14 concentrations in plasma were higher in AAA patients compared with controls, and deletion of CD14 reduced AAA formation in mice, suggesting a causal role for this innate immune signaling molecule in AAA pathogenesis (Blomkalns et al., 2013). Moreover, incubation of THP-1 monocytic cells with conditioned medium from PVAT resulted in upregulated CD14 expression and enhanced migration, suggesting that PVAT-derived pro-inflammatory cytokines may promote adventitial macrophage activation in AAAs.

Reactive oxygen species (ROS) are associated with vascular wall remodeling in AAAs (Emeto et al., 2016; Siu et al., 2017). Endothelin (ET)-1 plays a role in vascular ROS production and inflammation, and increased levels of ET-1 are associated with the formation of AAAs (Treska et al., 1999; Flondell-Sité et al., 2010). Li et al. reported that 8 weeks of high fat diet feeding induced AAAs in hyperlipidemic mice overexpressing ET-1 selectively in endothelium (eET-1) as compared to control mice. Levels of ROS and inflammatory cells (monocyte/macrophages and CD4 positive T cells) accumulating in PVAT were significantly higher in eET-1 mice than in control mice, suggesting that ET-1-dependent induction of oxidative stress and inflammatory responses in PVAT might contribute to AAA formation (Li et al., 2013).

**Figure 2:**
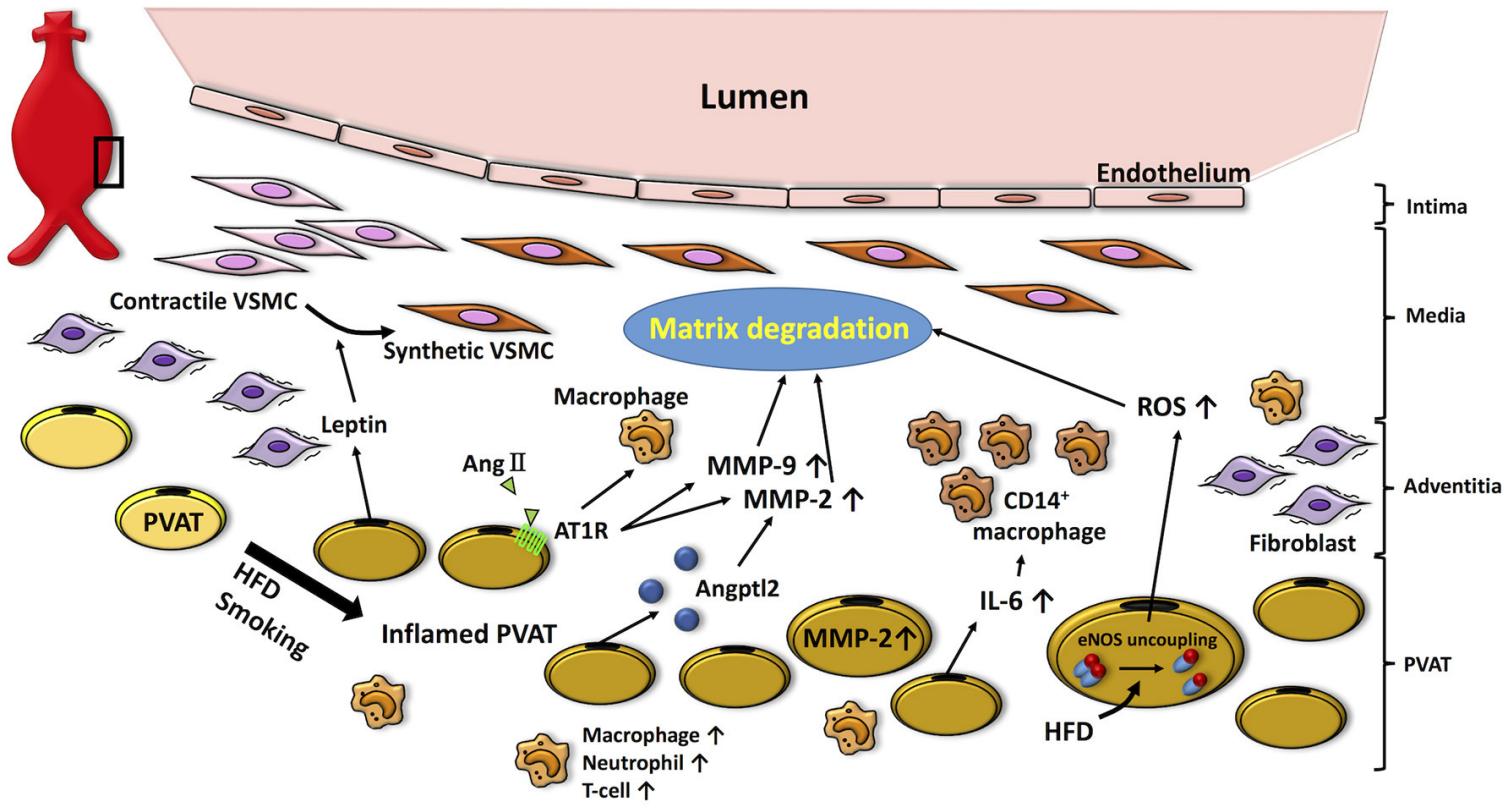
Potential role of PVAT in the pathogenesis of AAA. High fat diet- or smoking-induced PVAT inflammation promotes infiltration of inflammatory cells (i.e., macrophages, neutrophils, T cells), which facilitates matrix fragmentation through increases in MMP expression and activity. Moreover, adipocytokines (i.e., leptin) produced by PVAT induce VSMC phenotype switching associated with aneurysm disease. Inflamed PVAT also increases inflammatory cytokines (i.e., IL-6) and ROS, which further contributes to oxidative stress and matrix degradation in the vascular wall. HFD, high fat diet; AngII, angiotensin II; AT1R, angiotensin II type 1a receptor; Angptl2, angiopoietin-like protein 2; MMP, matrix metalloprotenease; IL-6, interleukin-6; ROS, reactive oxygen species; VSMC, vascular smooth muscle cell.

Sakaue et al. investigated the impact of deletion of the angiotensin II type 1a (AT_1a_) receptor in visceral adipose tissue transplanted to the abdominal aorta on AAA formation in hyperlipidemic mice (Sakaue et al., 2017). The authors transplanted 50 mg of visceral (epididymal) adipose tissue from wild-type or AT_1a_ knockout mice fed a high fat diet to the peri-abdominal aorta of recipient mice, which were then infused with AngII. AAA formation was markedly attenuated in mice that were transplanted with adipose tissue lacking AT_1a_ expression compared with control. Activities of MMP-2/MMP-9, and accumulation of F4/80-positive macrophages, were significantly lower than in mice transplanted with visceral adipose tissue from wild-type mice, suggesting that AT_1a_ receptor expression in PVAT can promote macrophage accumulation and MMP activity leading to AAA formation. However, a sham control group was not included in the study, so it is not possible to determine whether adipose tissue transplantation alone modulated AAAs. Moreover, epididymal adipose tissue rather than authentic PVAT was transplanted; thus, further studies are needed to clarify the direct effects of PVAT transplantation on AAA formation. Based on the available data, the potential role of PVAT in AAAs is illustrated in Figure 2.

## PVAT and arterial stiffness

Arterial stiffness is an independent predictor of cardiovascular events, such as heart failure, myocardial infarction, stroke, and kidney dysfunction (Hashimoto and Ito, 2011, 2013; Karras et al., 2012; Kitzman et al., 2013). Increased risk of cardiovascular disease with aging and the metabolic syndrome is in part attributable to arterial stiffening, as assessed by aortic pulse wave velocity in the clinical setting. Aortic stiffness is predominately determined by the balance and cross-linking status of extracellular matrix proteins, such as collagen and elastin, in the arterial wall (Prockop and Kivirikko, 1995; Debelle and Tamburro, 1999). Collagen type 1 is a key load-bearing collagen isoform whose increased expression is positively associated with aortic stiffness. Conversely, expression of elastin, a protein that provides elasticity to arteries, is inversely associated with aortic stiffness. Data in humans indicate that aortic stiffness is positively correlated with the quantity of associated PVAT, independent of body-mass index (Britton et al., 2013).

Experimental studies have begun to elucidate potential mechanisms whereby PVAT may modulate vascular stiffness. IL-6 is a pro-inflammatory cytokine secreted in higher concentrations from PVAT compared with other fat depots, and PVAT-derived IL-6 is associated with an increase in aortic stiffness. In addition, IL-6 concentration is related to pulse wave velocity in humans (Schnabel et al., 2008). Du et al. investigated whether aortic PVAT from hyperlipidemic mice promotes aortic stiffness and remodeling via PVAT-derived IL-6 secretion (Du et al., 2015), assessing intrinsic mechanical stiffness in aortic segments *in vivo* by aortic pulse wave velocity and by *ex vivo* techniques. Compared to wild-type mice, the hyperlipidemic mice exhibited increased aortic pulse wave velocity and intrinsic mechanical stiffness that was associated with higher expression of collagen type 1 and advanced glycation end products. Importantly, IL-6 secretion from PVAT of hyperlipidemic mice exceeded that of wild-type mice, and the enhanced intrinsic mechanical stiffness was reversed by a neutralizing antibody to IL-6 in hyperlipidemic mice. These data suggest that dysfunctional PVAT can promote intrinsic mechanical stiffening and matrix remodeling via enhanced IL-6 secretion.

Deficiency of lysyl oxidase, a copper-dependent amine oxidase, promotes fragmentation of elastic fibers (Chen et al., 2013). Chen et al. reported that mediators released from PVAT attenuate lysyl oxidase activity, thereby promoting elastin fragmentation and aortic stiffening. Thus, PVAT accumulation potentially may impair elastic fiber stability via several mechanisms to contribute to aortic stiffness.

To investigate the modulatory role of PVAT in arterial stiffness, PVAT was transplanted from young mice (4–6 months), old mice (26–28 months), or old mice treated with tempol (a superoxide dismutase mimetic) onto the abdominal aorta of young recipient mice (Fleenor et al., 2014). Eight weeks post PVAT transplantation, the young recipient mice transplanted with PVAT from old mice had greater aortic stiffness *in vivo* (increased aortic pulse wave velocity) and *ex vivo* (intrinsic mechanical stiffness) compared with those transplanted with PVAT from young donors. The PVAT of old mice contained higher levels of superoxide as compared with that of young mice, and tempol both normalized superoxide levels and abolished the enhanced aortic stiffness caused by transplantation of aged PVAT. Mechanistically, transplantation of aged PVAT led to increased expression of adventitial collagen type 1, which was likewise normalized by tempol treatment. These findings suggest that oxidative stress in PVAT can promote age-related aortic stiffness via the increased expression of collagen type 1, which potentially can be abrogated by targeted antioxidant therapy to PVAT.

## PVAT and vasculitic syndromes

Vasculitides represent a heterogeneous group of complex disorders characterized by acute and chronic inflammatory lesions of the vascular wall. As in both atherosclerosis and AAAs, macrophage infiltration is a critical component of vasculitides (Hansson and Hermansson, 2011), and is especially prominent in the adventitia. Granuloma formation is a typical finding in several vasculitides (Hilhorst et al., 2014). Lymphocytes and dendritic cells are enriched in these granulomas, which also contain neutrophils, eosinophils, and B cells. Infiltration of inflammatory cells within PVAT has also been demonstrated in vasculitic syndromes (Jakob et al., 1990; Wagner et al., 1996; Hollan et al., 2008). While several studies have demonstrated an association between PVAT and various vasculitides, direct evidence of a pathogenic role of PVAT is lacking, however.

Takayasu arteritis (TA) is a primary inflammatory disease of large elastic arteries such as the aorta and its major branches, with prominent accumulation of granulomas. TA is prevalent in young women and is more frequent in Asian countries compared to Europe and North America (Richards et al., 2010). The disease is characterized by an acute phase of constitutional symptoms followed by a vascular phase leading to arterial stenosis, occlusion and occasionally aneurysm formation. TA is diagnosed by typical clinical features and demonstrated narrowing of the aorta or its branches near their origin by conventional CT or CT angiography, magnetic resonance angiography, or ultrasonography (Choe et al., 2000; Gotway et al., 2005). Autopsy studies have described the inflammatory lesions as a nodular fibrosis that develops in the media and adventitia, extending into the *vasa vasorum*, along with intimal thickening (NASU, 1963; Hotchi, 1992; Matsunaga et al., 1998). Interestingly, TA patients have a greater prevalence of metabolic syndrome and higher levels of leptin and resistin, associated with upregulated inflammatory markers such as pentraxins-3 (Kawanami et al., 2004), compared to age-matched controls (Yilmaz et al., 2012; da Silva et al., 2013). Pentraxin-3 is considered to be a reliable activity marker of TA (Dagna et al., 2011). Lower levels of adiponectin were detected in TA patients with metabolic syndrome compared with those without metabolic syndrome. Given that the vessels afflicted with TA are highly endowed with PVAT, it is tempting to speculate that PVAT may contribute to local inflammation of the vascular wall in TA, but definitive evidence is lacking. Analysis of CT scans in patients with TA to quantify the FAI in PVAT could help to define its utility as a marker of PV inflammation and local disease activity in these patients.

## Conclusions

A growing body of data support linkages between PVAT and non-atherosclerotic vascular diseases, including neointimal hyperplasia, aortic aneurysm, arterial stiffness, and vasculitic syndromes, in experimental animal models and in humans. These diseases are all associated with vascular wall inflammation that may be modulated by adipocytokines produced by PVAT. Moreover, mediators produced by PVAT can also regulate VSMC proliferation, matrix degradation, and neovascularization associated with these diseases. The extent to which PVAT is a mediator vs. a marker of non-atherosclerotic vascular disease, or even a promoter of vascular health, remains to be determined. The commonly employed animal models used to examine the role of PVAT in vascular diseases suffer from technical limitations and confounding variables that may complicate data interpretation. Development of more robust animal models is clearly needed to advance our understanding of the role of PVAT in non-atherosclerotic vascular diseases. Exciting new data in humans suggest that imaging PVAT may prove useful to uncover its potential role in vascular diseases and perhaps even to direct new therapeutic approaches.

## Author contributions

All authors listed, have made substantial, direct and intellectual contribution to the work, and approved it for publication.

### Conflict of interest statement

The authors declare that the research was conducted in the absence of any commercial or financial relationships that could be construed as a potential conflict of interest. The reviewer JP and handling Editor declared their shared affiliation.
